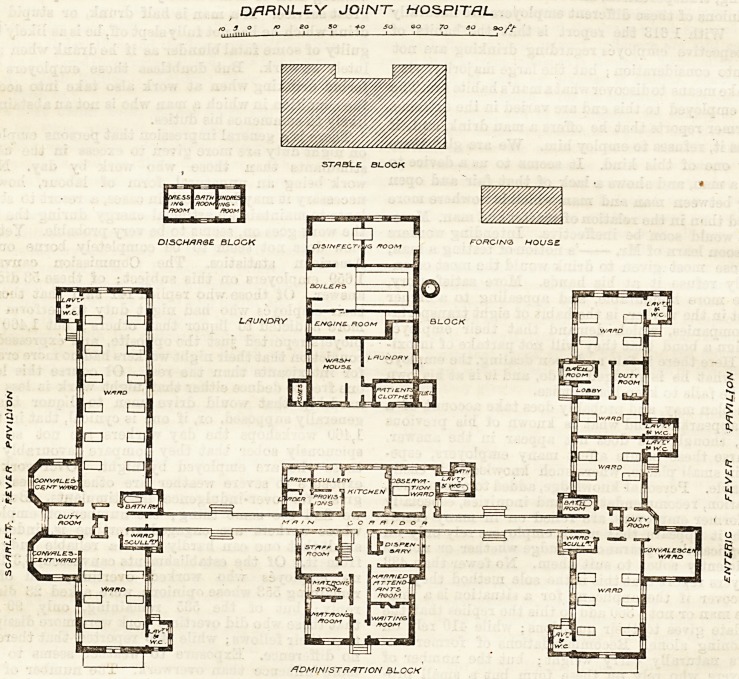# Hospital Construction

**Published:** 1898-12-24

**Authors:** 


### The Institutional Workshop.

### HOSPITAL CONSTRUCTION.

### HOSPITAL FOR INFECTIOUS DISEASES, DARNLEY.

The plans of this building, which was designed by Mr. J. L. Cowan, and opened at the end of last year, provide accommodation for 42 patients, arranged in two pavilions standing on either side of the administration block, with which they are connected by open corridors. In the rear there are four separate blocks, respectively designed for (*a*) the discharge of patients, (*b*) stables for the ambulance, &c., (*c*) engine-room and laundry, and (*d*) a conservatory for the supply of plants to the wards. The pavilion on the left is devoted to scarlet fever cases, and that on the right mainly to enteric cases, with a smaller sub-division for receiving patients suffering from other diseases requiring isolation. The plan is designedly compact, and the bath rooms and w.c.'s are not as distinctly separated from the wards as is usual in larger fever hospitals. The position of the convalescent wards, opening in one case from the duty room, and in the others from the large wards, is also little open to question, while the observation ward is next the kitchen, and appears to be too much a part of the administrative block. The upper floor of the latter, not shown on the plan, contains eight bedrooms and a bath-room for the use of the staff, while there is also, in the grounds of the hospital, a separate house for the resident engineer.

An interesting feature of the design is the mechanical ventilation of the wards, each pavilion being supplied by an electrically-driven fan with air drawn through a wooden screen from a height of five feet above the ground. The screen is kept wet, and the air afterwards passes over steam-pipes which give it the necessary warmth in winter. The heating of the hospital is effected by this current of air, which is conveyed to the wards through sheet-iron ducts wrapped with non-conducting material. The engineering side of the arrangements, designed by Mr. Thomas Young, appears to be very complete, including steam disinfectors, laundry machinery, electric lighting, telephones, and other labour-saving appliances. The total cost has been £357 per bed, which compares favourably with that of many similar institutions recently erected.

## Figures and Tables

**Figure f1:**